# Treatment of Central Nervous System Infection Caused by Multidrug-Resistant *Klebsiella pneumoniae* with Colistin Sulfate Intravenously and Intrathecally: A Case Report

**DOI:** 10.3390/ph15121482

**Published:** 2022-11-29

**Authors:** Xin Lu, Cejun Zhong, Haifeng Chen, Xiaoqi Xie, Xiaoju Lv

**Affiliations:** 1 Center of Infectious Diseases, West China Hospital of Sichuan University, Chengdu 610041, China; 2 Neurosurgery Department, West China Hospital of Sichuan University, Chengdu 610041, China; 3 Neurological Intensive Care Unit, West China Hospital of Sichuan University, Chengdu 610041, China

**Keywords:** colistin sulfate, central nervous system infection, cerebrospinal fluid, multidrug-resistant *Klebsiella pneumoniae*, infection foci

## Abstract

**Background**: Due to the blood–brain barrier and limited antibiotic choices, polymyxin is currently the first-line agent for the treatment of central nervous system infections (CNSIs) caused by multidrug-resistant Gram-negative bacteria (MDR-GNB). Colistin sulfate, as a polymyxin E different from CMS, is used in Chinese clinics, and there are limited reports on its use in the treatment of CNSIs. **Case Presentation:** This case describes a 76-year-old man who underwent complex neurosurgery for cervical spinal stenosis. Postoperatively, the patient developed a fever and a poorly healed surgical wound. Numerous blood routine tests, inflammatory markers, pathogenic tests of cervical secretions, cerebrospinal fluid (CSF), and sputum were sent for diagnosis. After empirical antimicrobial treatments failed, the CSF and wound pus cultured carbapenem-resistant *Klebsiella pneumoniae*. The regimen was adjusted to colistin sulfate intravenously and intrathecal injection combined with tigecycline. In addition, the management of infection foci, including continuous lumbar pool drain, cervical 3–5 internal fixation removal with cervical 1–6 spine dilation, CSF leak repair, and right thigh broad fasciotomy, were performed. After treatment, the patient was discharged with multiple sets of negative CSF cultures and the infection under control. **Conclusions:** For CNSIs caused by MDR-GNB, the selection of colistin sulfate for intravenous and topical combination treatment is a viable choice.

## 1. Introduction

Multidrug-resistant organisms (MDROs) are pathogenic bacteria that are resistant to three or more routinely used antimicrobial agents. Neurosurgical central nervous system infections (NCNSIs) are a common postoperative complication of neurosurgery. In recent years, NCNSIs caused by Gram-negative bacteria have been rising. Polymyxin-based combination therapy is the first-line treatment for NCNSIs caused by multidrug-resistant Gram-negative bacteria (MDR-GNB) [1,2]. There are three types of polymyxins available for clinical application, including colistimethate sodium (CMS), polymyxin B sulfate (PBS) and colistin sulfate (CS).

CMS is an inactive precursor drug that must be transformed into active polymyxin E in the body. It is predominantly processed by the kidneys [3], and its conversion in the cerebrospinal fluid (CSF) remains unknown. PBS and CS act directly with no need for conversion in vivo, being metabolized mainly via non-renal routes. Common adverse effects include nephrotoxicity, neurotoxicity, and pigmentation of the skin (polymyxin B sulfate only). Several studies have shown the efficacy of intravenous infusions of CMS or PBS and intraventricular/intrathecal injections in the treatment of NCNSIs [4,5,6]. CS (from Shanghai New Asia Pharmaceuticals, Shanghai, China) was approved for clinical use by the Chinese National Medical Products Administration on 16 April 2018 [7], and has rarely been reported in its clinical application. In this paper, we report a case of systemic and topical CS-based medication combined with surgical intervention for the treatment of CNSI due to multidrug-resistant *Klebsiella pneumoniae* after neurosurgery, aiming to provide a reference for the anti-infective treatment of NCNSIs due to MDR-GNB.

## 2. Case Report

This is a 76-year-old male with a BMI of 38.06 kg/m^2^. He acquired numbness in his upper limbs for no apparent reason three months ago. Before twenty days, he felt that the numbness in his upper limbs had worsened and was accompanied by stiffness, weakness, and unstable walking. A head, neck, and lumbar MRI was performed at the Hospital of China MCC5 Group Corporation and cervical marrow compression was found; thus, the patient was transferred to the West China Hospital of Sichuan University. He has a history of hypertension with an undetermined maximum blood pressure that is effectively managed by nifedipine 20 mg bid. He endured an “intravertebral lipoma resection” over a decade ago. Sixty years ago, he also underwent a “right inguinal hernia procedure”.

The patient was awake upon admittance. Positive signs were grade IV muscle strength in the limbs, instability when grasping objects in the upper limbs, decreased sensation in the upper limbs and trunk with a feeling of banding, and failure to perform the bilateral finger–nose test.

**Figure 1:**
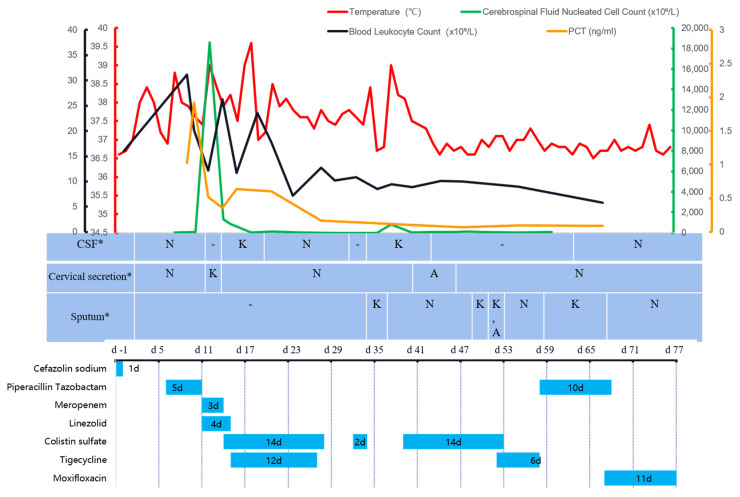
The temperature, cerebrospinal fluid nucleated cell count, blood leukocyte count, PCT, culture results of specimens sent, and the use of antimicrobials during hospitalization. Description: PCT: procalcitonin; CSF: cerebrospinal fluid; K: carbapenem-resistant *Klebsiella pneumoniae*; A: carbapenem-resistant *Acinetobacter baumannii*; N: unknown term; -: negative. d-1: day-1; 10 February 2022, is day 0. *: The time corresponding to the table is the time the specimen was sent for testing, and the culture generally returns results in 3–5 days.

The preoperative examination showed that the white blood cell count was 16.03 × 10^9^/L (Figure 1), predominantly neutrophil granulocytes, accounting for 76.5%. Preoperative creatinine was 122 umol/L, fasting blood glucose was 7.2 mmol/L, and liver function and coagulation function were normal. The MRI of the neck showed a soft tissue shadow in the left intervertebral foramen area of cervical 2/3, with a high probability of neurogenic tumor; partial bony defects in the left arch of cervical 2 and 3, degenerative changes in the cervical spine, herniated discs in cervical 3–7 and cervical spinal stenosis; and an abnormal signal on the right side of the cervical medulla, the nature of which was unknown. On 10 February 2022 (day 0), the patient underwent general anesthesia for “excision of occupying lesions in cervical 2 and 3 spinal canals, decompression of cervical 1–5 spinal nerve roots, decompression of cervical 1–5 laminae, internal fixation of cervical 3–5 lateral block screws with bone graft fusion, and repair of cervical 2–3 CSF leak”. There was no preoperative fever and cefazolin sodium (1 g q12 h, one dose preoperatively, and one dose 24 h postoperatively) was given for prophylaxis. A scar from a prior surgical incision was observed intraoperatively in the posterior neck, with no abnormalities in the dermato-muscular region; the atlantoaxial spine was narrowed, the cervical 3–5 spinal canal was narrowed, and the dura was considerably compressed.

After surgery, it was difficult to remove the ventilator from the patient. On day 1, a fever with a peak temperature of 38 °C (Figure 1) emerged, which was initially managed with antipyretic symptomatic medication. On day 5, the tracheal tube was extracted. On day 6, the patient with persistent fever underwent a lumbar puncture, and a lumbar pool drainage tube was inserted. The results of the CSF examination are depicted in Figure 1. The CSF was yellowish and slightly cloudy. Piperacillin/tazobactam (4.5 g q8 h) was experimentally provided to treat the extracranial infection based on the test results. The patient’s condition worsened on day 8 due to gastrointestinal bleeding, low blood pressure, and abnormal liver and renal function. The patient was subsequently transferred to the neurological intensive care unit (NICU). With a maximal temperature of 39 °C, the patient’s hyperthermia persisted. With CSF leakage, the cervical incision dressing was still oozing and emanating a foul odor. Piperacillin/tazobactam was substituted with meropenem (2 g q8 h) and linezolid (600 mg q12 h) due to CSF leaking and central nervous system infection. On day 14, the patient’s oxygen saturation gradually fell, which was attributed to his deteriorating neck wound infection, and he was extubated with a Glasgow Coma Scale (GCS) score of E1VTM1, indicating a severe consciousness disorder. In the interim, cultures of cervical secretions and cerebral fluid consistently revealed carbapenem-resistant *Klebsiella pneumoniae* (CRKP) (drug susceptibility results are shown in Table 1). On day 14, the postoperative incision was reinforced with sutures and dressing changes. Additionally, on the same day, the treatment regimen was changed to CS (750,000 U iv q12 h plus 50,000 U intrathecal injection QD), combined with tigecycline (50 mg q12 h, discontinued 12 days later) against CRKP infection.

The patient continued to experience recurring fever with a high temperature of 39.6 °C after the regimen adjustment. The site of the lumbar pool was relocated on day 17. On day 18, the internal fixation removal of cervical 3–5 vertebrae, cervical 1–6 vertebrae dilatation, CSF leak repair, and right thigh broad fasciotomy were performed. The next day, the intrathecal infusion of CS was modified to 50,000 U qod. On day 22, due to a severe infection, protracted tracheal intubation and obesity, the patient had a tracheotomy under general anesthesia. On day 26, the patient’s consciousness improved, as indicated by a GCS score of E3VTM4 and improved blood tests. The patient was transferred back to the general ward the following day, and CS was withdrawn. On day 31, following the infectious disease physician’s consultation, CS was again delivered intravenously (500,000 U q12 h). Due to economic difficulty, the patient’s family declined to utilize the medication. On day 37, the patient’s temperature reached a maximum of 39 °C. The postoperative cervical incision was not healing well, and the bacterial culture of the CSF and sputum still exhibited CRKP. After explaining to the family member the necessity of using sensitive antimicrobial drugs due to the patient’s condition, and informing them of the health insurance policy for the drugs involved, the patient was restarted on intravenous CS (500,000 U q12 h). The lumbar pool drainage was continued with approximately 150–250 mL of CSF daily, and the tube was replaced once more on day 40.

**Figure 2:**
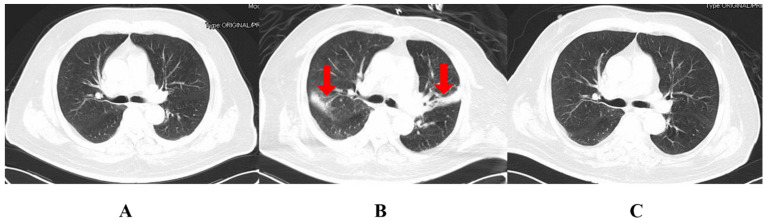
The CT of the chest during the patient’s hospital stay. (**A**): the Chest CT on 8 February (two days before surgery); (**B**): the Chest CT on 4 March (day 22); (**C**): the Chest CT on 22 April (day 71). The areas indicated by the red arrows are the sites of infection.

The patient’s temperature returned to normal after the repeated administration of CS on day 37 (Figure 1). Multiple sputum cultures with CRKP and carbapenem-resistant Acinetobacter baumannii (CRAB) were performed (Table 1). However, leukocytes and inflammatory indices eventually reverted to normal and dropped. A chest CT (Figure 2) indicated considerable resorption of the pulmonary lesion, and MDRO colonization was considered at this point. The cerebral fluid was re-examined on a regular basis (Figure 1), and the number of nucleated cells and microproteins gradually decreased. On day 51, CS was discontinued and tigecycline was introduced in its place. Step-down therapy was continued with a switch to piperacillin/tazobactam and a stepwise sequence to oral moxifloxacin, given the satisfactory control of the patient’s infection. A culture of neck wound secretions showed CRKP on day 40 and 43, but the secretions were significantly reduced. The lumbar pool drain and tracheal tube were withdrawn on day 62 and day 63, respectively. The illness was then stabilized, oral antibiotics were administered, and the patient was discharged. During treatment with CS, the patient experienced a transient hypercreatinemia (from 122 to 188 umol/L), which decreased to a normal level during later treatment with the drug.

## 3. Discussion

The vulnerable factors for NCNSI in this patient were as follows [10]: age > 70 years, poor glycaemic control, presence of an implant, postoperative wound CSF leak, and the presence of subcutaneous fluid in the surgical incision. Combined with the history of implant surgery, recurrent hyperthermia, ancillary tests, and pathogenic findings in the CSF, the diagnosis of CNSI was clear. Due to his poorly healed surgical wound, postoperative CSF leakage from the incision site, and wound secretions consistent with drug-sensitive results of CSF pathogens, it was considered that the CRKP of intracranial infection originated from a prolonged postoperative neck incision infection. CRKP is a carbapenem-resistant Enterobacteriaceae (CRE) bacterium. It produces enzymes that hydrolyze carbapenems, resulting in carbapenem resistance. The widespread resistance mechanism of CRKP is the production of KCP carbapenamse; KCP-2, especially, is epidemic in our country [11]. In addition, typical carbapenemases include metallo-β-lactamases (MβLs) and OXA-type β-lactamases, with the OXA enzyme being more commonly seen in CRAB [12,13].

In the empirical treatment of CNSIs, antimicrobial medications need to cover prevalent pathogenic bacteria considering the regional epidemiology. In this case, broad-spectrum antibiotics of piperacillin/tazobactam were initially employed against Gram-positive and -negative bacteria as well as anaerobes because of the CSF results suggesting insufficient evidence of intracranial infection. However, when the patient’s infection deteriorated, the postoperative incision healed poorly, and a CSF fistula occurred, the antibiotic treatment was escalated to meropenem for meningitis caused by Gram-negative bacteria typical of nosocomial infections and linezolid for Gram-positive bacteria. Later, the bacteria cultured from the CSF were identified as CRKP.

The treatment options should be chosen based on the drug sensitivity results once the culture results indicate MDR-GN, and there are relatively few medications available for the treatment of NCNSIs. High-dose meropenem is the first-line treatment for GN that produces extended-spectrum beta-lactamase and is sensitive to carbapenems in vitro [1]. The limited injectable treatments for carbapenem-resistant GN are aminoglycosides, polymyxins and novel β-lactam/β-lactamase inhibitors [14]. For the treatment of NCNSIs, intravenous high-dose administration is not applicable for all of the above medications. As an alternative, drugs can be injected directly into the brain’s ventricles without having to cross the blood–brain barrier. In this case, CRKP were susceptible to tigecycline, ceftazidime/avibactam and polymyxins (shown in Table 1). Intravenous tigecycline has a low concentration in the CSF. According to some studies [15,16], tigecycline can be administered intravenously and ventricularly to treat CNSIs caused by CRAB. However, Li et al. [16] documented instances in which tigecycline may have triggered spinal arachnoiditis in patients. The efficacy and safety of tigecycline need to be further evaluated. In addition, the international consensus [1] for the treatment of CNSIs does not include tigecycline as a recommended drug for intracerebroventricular injection. The Chinese Expert Consensus on the Diagnosis, Treatment, and Prevention of Carbapenem-Resistant Enterobacteriaceae Infections [17] also does not recommend tigecycline for the treatment of CNSIs due to CRE. Ceftazidime/avibactam, as a novel β-lactam/β-lactamase inhibitor, has had limited clinical studies for the treatment of CNSIs. In the rabbit meningitis model [18], the average penetration rate of avibactam in the CSF was 38%. Its efficacy has yet to be accumulated clinically.

According to the 2021 China Antimicrobial Surveillance Network (CHINET), 9.1% of patients with intracranial infections had *Klebsiella pneumoniae* in their CSF. CRKP had the lowest rate of polymyxin resistance, with a lower rate of polymyxin E resistance (5.3%) than polymyxin B (5.8%). Polymyxin, as a cationic peptide antibiotic, destabilizes the outer membrane of Gram-negative bacteria by binding to negatively charged lipopolysaccharides (LPS), which destroys the outer membrane of the bacteria and causes the cytoplasmic contents to lyse, resulting in the death of the bacteria [19]. As a large molecule, polymyxin is difficult to achieve effective concentrations in the CNS with intravenous administration alone. Increasing the intravenous dose increases the prevalence of adverse events, necessitating topical coadministration [1]. In China, PBS, CS and CMS for injection were launched in 2017, 2018 and 2020, and their prices are also decreasing in order. However, only the first two are currently included in category B of China’s medical insurance, in which the patient pays a certain percentage of the cost of the drug, and the rest is covered by basic medical insurance and reimbursed proportionally. Combining pharmaco-economics and medicine availability, this patient was treated with CS against infection.

Indeed, there have been many clinical studies and drug trials reported on colistin, but the vast majority of colistin refers to CMS, and only a small percentage of non-clinical studies based on CS. Although both of them are polymyxin E, CMS is not the same as CS. CMS has a delayed bactericidal effect in vivo compared to active CS. Due to the conversion and predominantly renal metabolism of CMS [3], high doses are not only required to reach effective therapeutic concentrations, but also increase the risk of renal damage [1]. Therefore, the terms of colistin sulfate and CMS should not be interchanged.

Since the clinical use of CS, few studies have been reported on its use in CNSIs. Yu et al. [20] reported a case of NCNSIs caused by multidrug-resistant *Acinetobacter baumannii*, in which the intravenous administration of CS combined with intraventricular injection resulted in the significant improvement of ventriculitis without adverse effects such as nephrotoxicity and neurotoxicity. In another case of CRAB-induced NCNSI treated with the intrathecal and intravenous administration of CS, Cheng et al. [21] reported that the patient experienced hallucinations, babbling, and irrelevant responses during administration, which were attributed to the neurotoxicity of CS in combination with meropenem. After adjusting the frequency of the ventricular administration of this drug, the patient’s consciousness gradually improved. The CNSI suffered by the patient, in this case, was caused by CRKP and a transient increase in creatinine was observed during drug administration. In the context of the patient’s clinical condition and laboratory investigations, it was considered that the creatinine increase was associated with the inflammatory injury of uncontrolled severe infection. Upon continuing treatment with CS, the creatinine level returned to normal. No adverse effects such as neurotoxicity or skin pigmentation have been observed in this patient.

In this instance, the intravenous dose of CS was 750,000 U every 12 h, and the intrathecal dose was 50,000 U daily for three days, and then every other day. Because of the recent availability of CS for intravenous use, there is no recommended dose for its intrathecal administration. Given that the intravenous pharmacokinetics parameters of CS and PBS are similar [22], the intrathecal dosing of CS was determined empirically using the recommended dose units for intrathecal PBS. However, the optimal dose for intrathecal CS treatment requires further investigation. In addition to CS, the patient’s neck wound and lung infections were treated with intravenous tigecycline antimicrobial therapy. Together with the surgical intervention of the infected site, the pathogen in the CSF was eradicated and the infection was finally brought under control.

After the patient was transferred from the neonatal intensive care unit to the general ward, the use of CS was discontinued due to the patient’s improved CSF results and the patient’s family’s financial situation. The consensus of polymyxins [23] suggests submitting CSF cultures daily or every other day for 10 to 14 days following the last positive culture before discontinuing the drug. Based on the consensus on CRE Infections in China [17], the duration of treatment for CNSIs is typically at least three to four weeks. According to the Chinese Expert Consensus on the Management of CNSIs in Neurosurgery (2021 Edition) [10], for severe CNSIs, a long course of treatment of 4–8 weeks should be administered, and antimicrobial therapy should be continued for 10–14 days after meeting the clinical cure criteria. According to the consensus, the initial course of CS, in this case, was insufficient, resulting in a recurrence of infection and a pathogen-positive CSF culture after drug discontinuation. The patient improved after receiving additional CS treatment. In the case described by Cheng et al. [21], the initial course of CS was insufficient and the drug was reintroduced. However, the duration of administration is inconsistent with consensus recommendations, and the available case reports of positive outcomes report varying durations of CS use, indicating the need for the individualization of antimicrobial use.

In reality, the therapeutic window for polymyxins is narrow, with therapeutic and toxicogenic doses nearly overlapping [23]. Therefore, therapeutic drug monitoring (TDM) is required which can assist physicians in monitoring therapeutic concentration attainment and adjusting drug doses accordingly. On the other hand, regular TDM can assist clinicians in determining when it is appropriate to discontinue a drug and reduce the patient’s financial burden. TDM was not, however, performed on this patient, and the current use of CS is still empirical. In addition, the patient was morbidly obese and had a high daily CSF drainage, making it impossible to determine the concentration of the therapeutic drug at the site of infection, resulting in a recurrence of infection after drug discontinuation. Consequently, TDM is essential for both patients and medical professionals and needs to be implemented in our hospital in the near future.

## 4. Conclusions

In conclusion, the antimicrobial regimen based on the intravenous infusion and intrathecal injection of CS combined with tigecycline is effective in the treatment of MDR-GNB-caused NCNSIs, wound infections, and lung infections without causing significant adverse effects. However, intrathecal CS dosing remains empirical, and drug concentrations need to be monitored throughout treatment. In addition, future multicenter, randomized clinical trials will be useful for evaluating its dosing, duration, safety, and efficacy.

## Figures and Tables

**Table 1:** The susceptibility results of antimicrobials against *Klebsiella pneumoniae* *.

Antibiotics	Methods	MICs	Results
STC	KB	+	+
AMO/CLA	MIC	≥32	R
Amikacin	MIC	≥64	R
Ampicillin	MIC	≥32	R
Aztreonam	MIC	≥64	R
Ceftazidime	MIC	≥64	R
Cefoxitin	MIC	≥64	R
Ciprofloxacin	MIC	≥4	R
Cefpodoxime	MIC	≥8	R
Ceftriaxone	MIC	≥64	R
CPZ/SBT	KB	7	R
Cefotaxime	MIC	≥64	R
Cefuroxime	MIC	≥64	R
CAZ/AVI	KB	21	S
Cefazolin	MIC	≥64	R
Donipenem	MIC	≥8	R
Gentamicin	MIC	≥16	R
Imipenem	MIC	≥16	R
Levofloxacin	MIC	≥8	R
Meropenem	MIC	≥16	R
Moxifloxacin	MIC	≥8	R
Minocycline	KB	11	R
Polymyxin B	MIC	0.5	WT
AMP/SBT	MIC	≥32	R
SMZ	MIC	≥16/304	R
TIC/CLA	MIC	≥128	R
Tetracycline	MIC	≥16	R
Tigecycline	MIC	1	S
Ticarcillin	MIC	≥128	R
Tobramycin	MIC	≥16	R
PIP/TAZ	MIC	≥128	R

KB: disc diffusion method. MIC: minimum inhibitory concentration. S: susceptible. R: resistant. WT: wild type. STC: serine-type carbapenemases. AMO/CLA: amoxicillin/clavulanic acid. CPZ/SBT: cefoperazone/sulbactam. CAZ/AVI: ceftazidime/avibactam. AMP/SBT: ampicillin/sulbactam. TIC/CLA: ticarcillin/clavulanic acid. PIP/TAZ: piperacillin/tazobactam. SMZ: sulfamethoxazole. *: the *Klebsiella pneumoniae* were extracted and identified using matrix-assisted laser desorption/ionization time-of-flight mass spectrometry (MALDI-TOF/MS). Via broth microdilution, antimicrobial susceptibility of *Klebsiella pneumoniae* to colistin sulfate was determined in accordance with experts’ consensus [8] based on the United States Committee on Antimicrobial Susceptibility Testing (USCAST) [9].

## Data Availability

Data is contained within the article.
